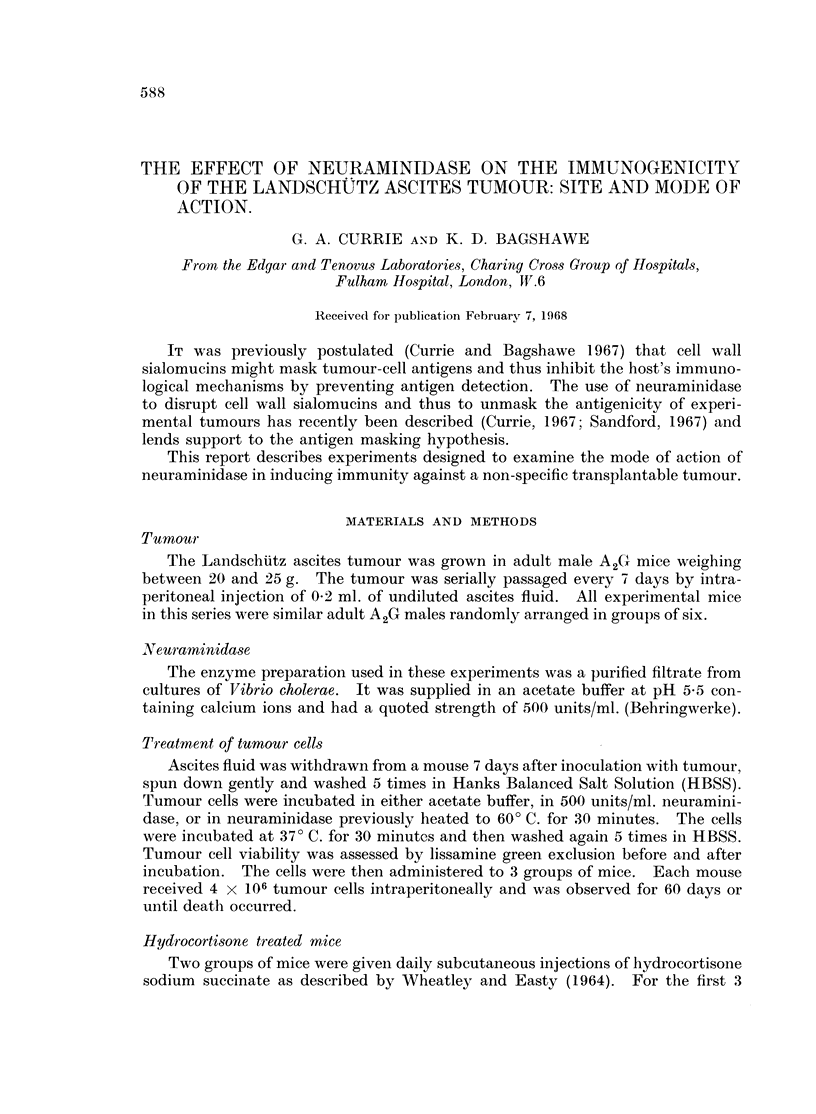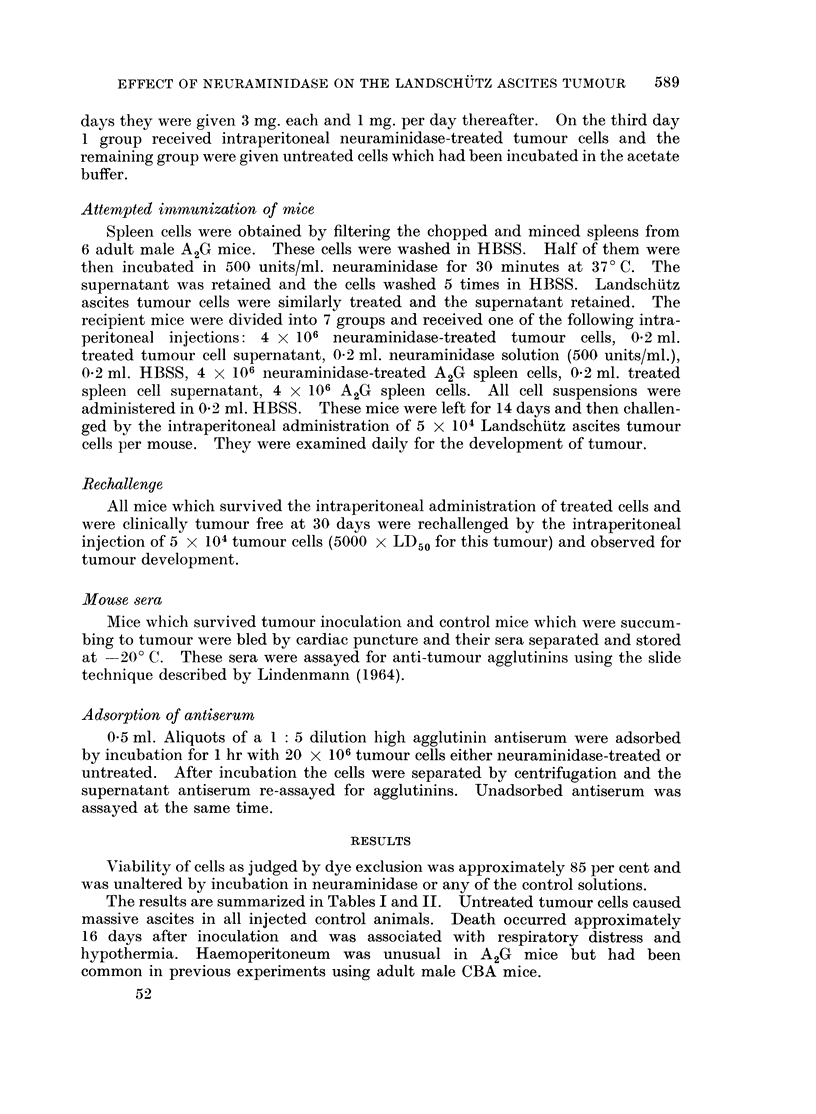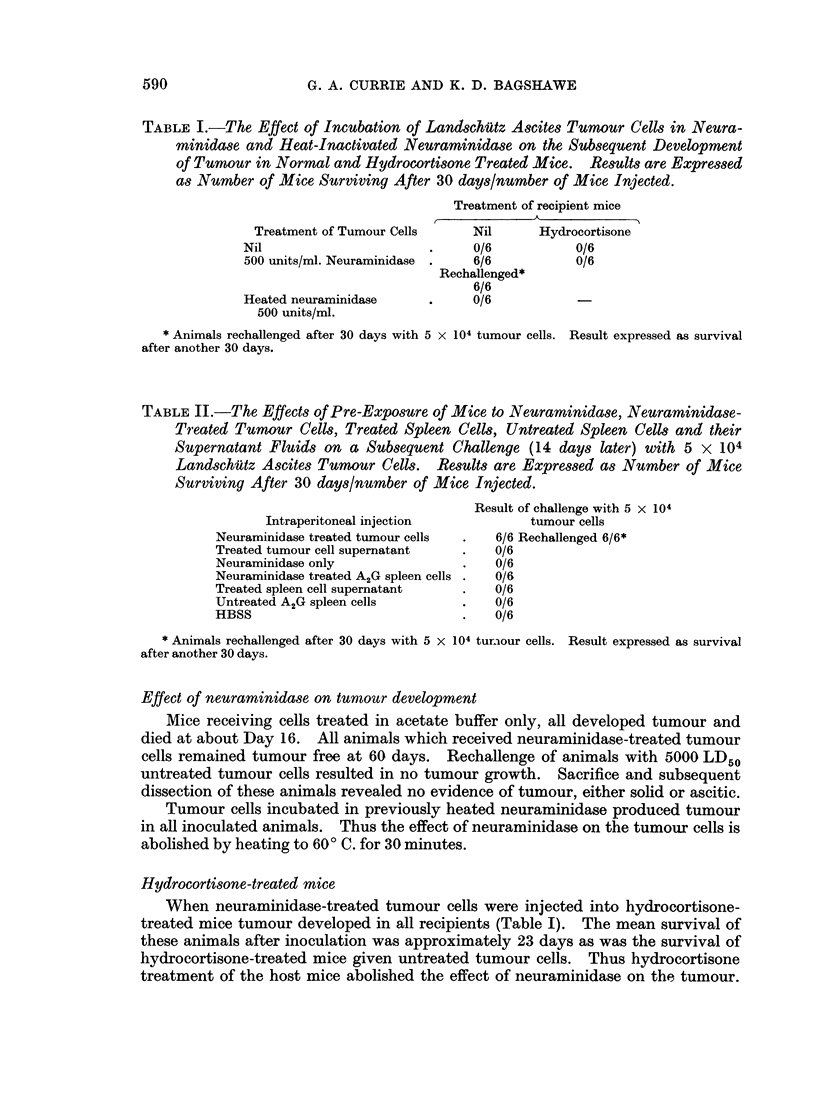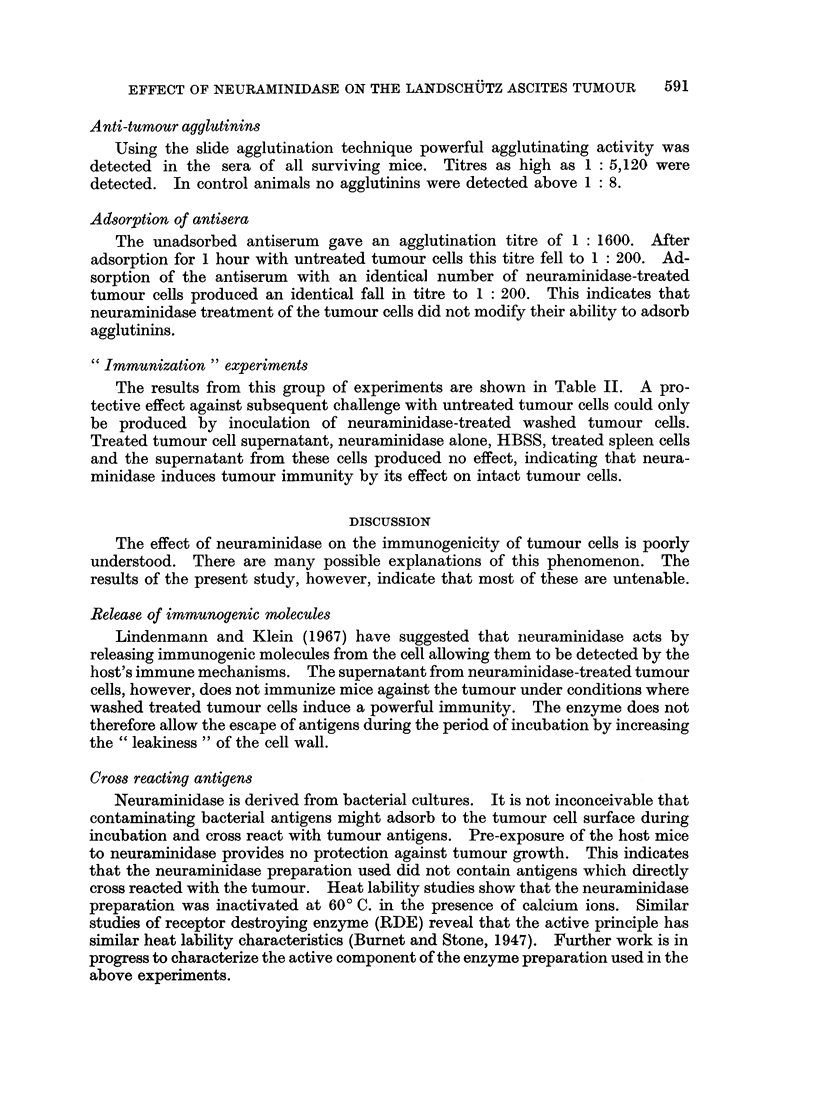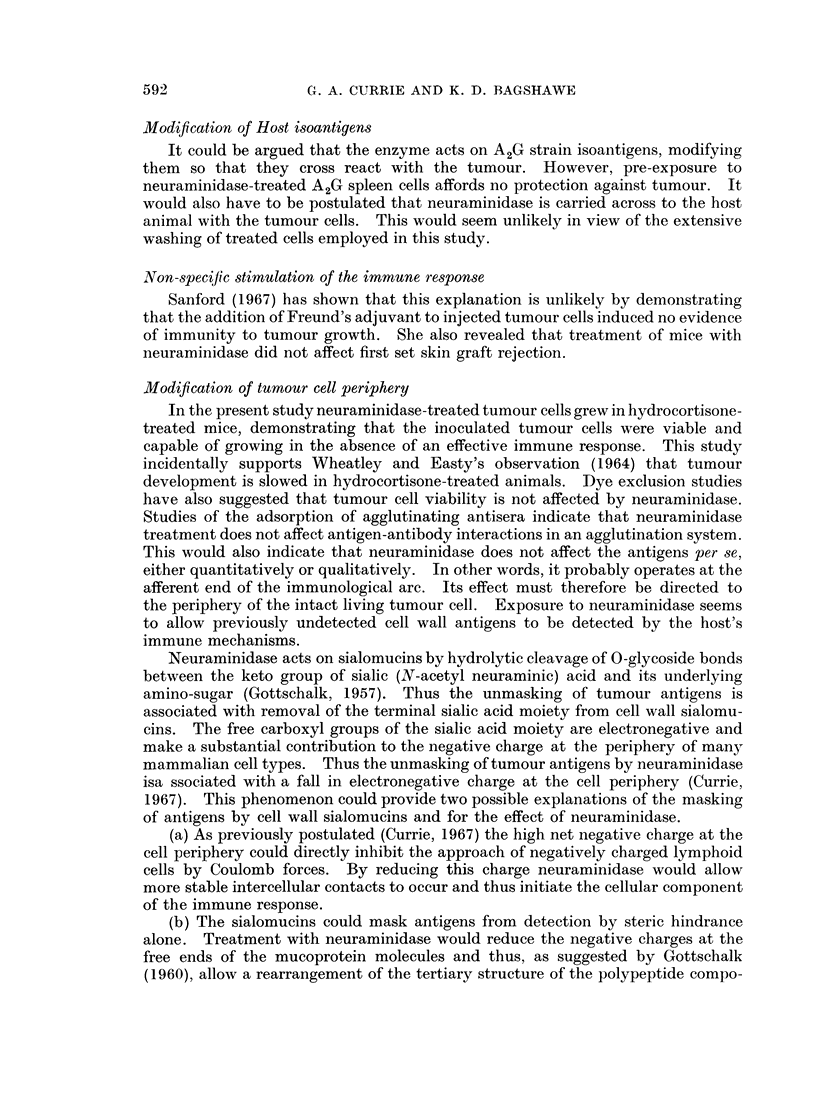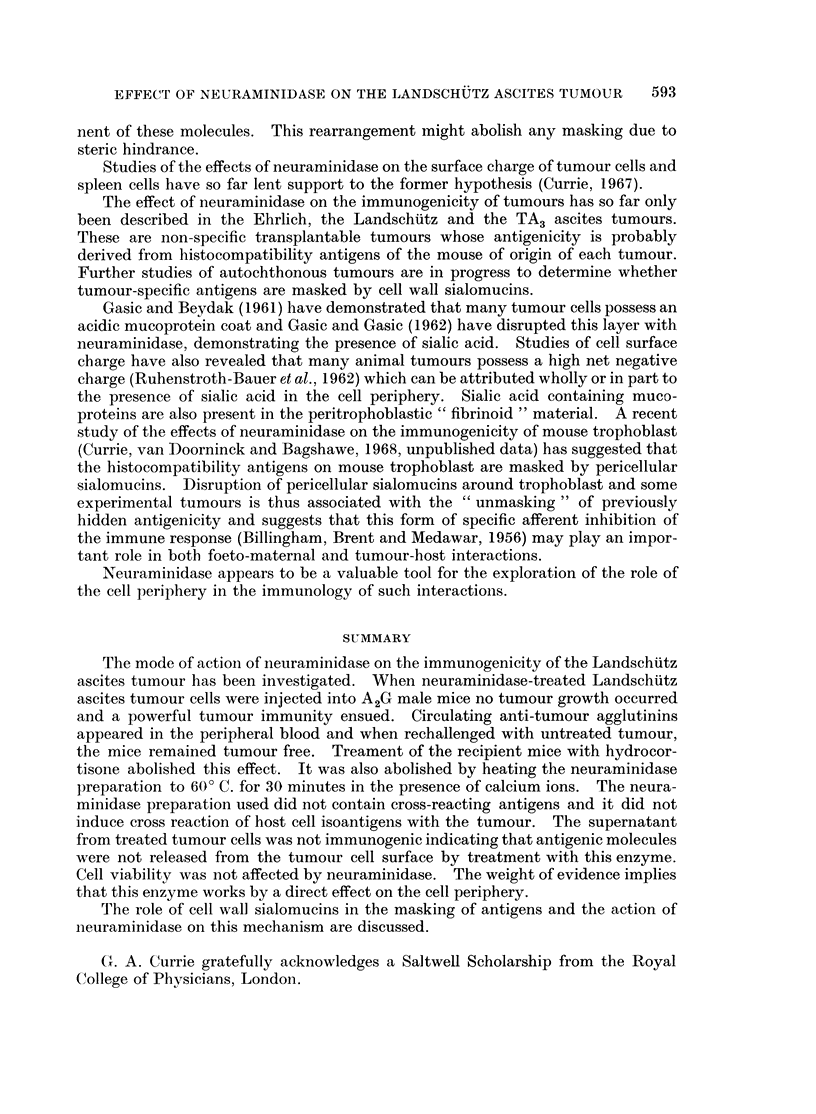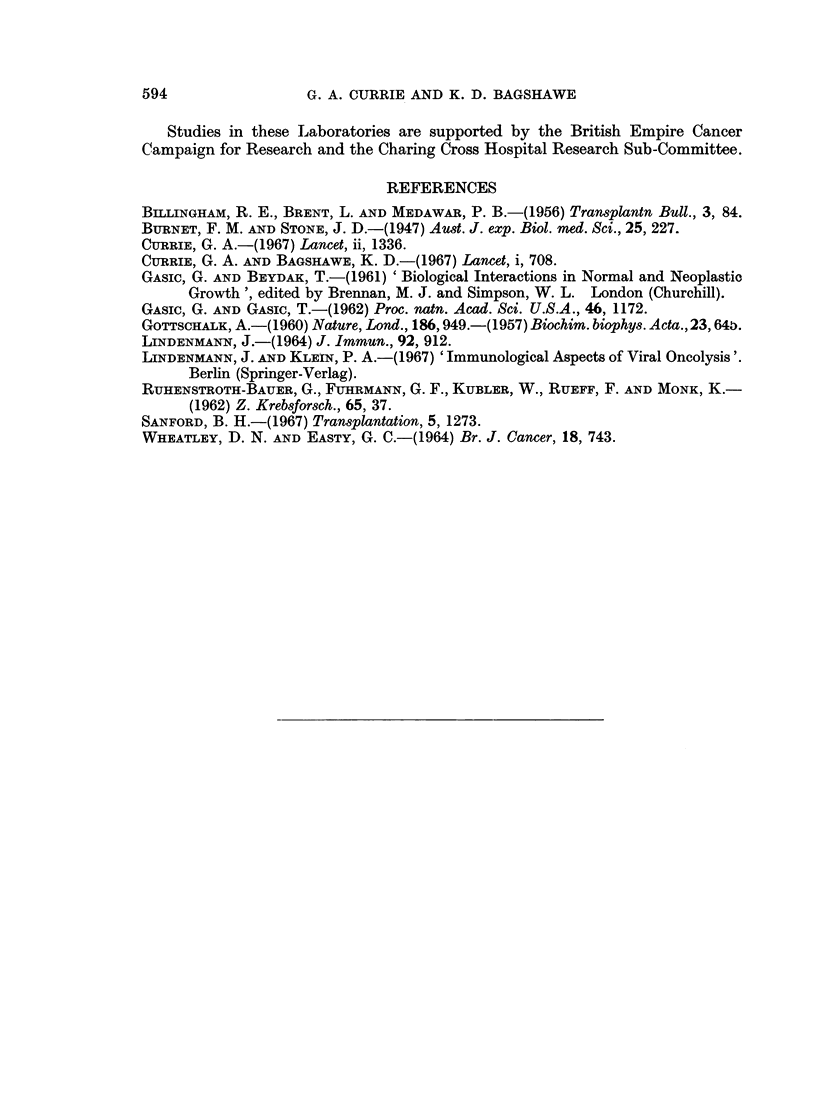# The effect of neuraminidase on the immunogenicity of the Landschütz ascites tumour: site and mode of action.

**DOI:** 10.1038/bjc.1968.70

**Published:** 1968-09

**Authors:** G. A. Currie, K. D. Bagshawe


### THE EFFECT OF NEURAMINIDASE ON THE IMMUNOGENICITY OF THE LANDSCHÜTZ ASCITES TUMOUR: SITE AND MODE OF ACTION.

G. A. CURRIE AND K. D. BAGSHAWE

*From the Edgar and Tenovus Laboratories, Charing Cross Group of Hospitals, Fulham Hospital, London, W.6*

Received for publication February 7, 1968

It was previously postulated (Currie and Bagshawe 1967) that cell wall sialomucins might mask tumour-cell antigens and thus inhibit the host's immunological mechanisms by preventing antigen detection. The use of neuraminidase to disrupt cell wall sialomucins and thus to unmask the antigenicity of experimental tumours has recently been described (Currie, 1967; Sandford, 1967) and lends support to the antigen masking hypothesis.

This report describes experiments designed to examine the mode of action of neuraminidase in inducing immunity against a non-specific transplantable tumour.

### MATERIALS AND METHODS

### *Tumour*

The Landschütz ascites tumour was grown in adult male $A_2G$ mice weighing between 20 and 25 g. The tumour was serially passaged every 7 days by intraperitoneal injection of 0·2 ml. of undiluted ascites fluid. All experimental mice in this series were similar adult $A_2G$ males randomly arranged in groups of six.

### *Neuraminidase*

The enzyme preparation used in these experiments was a purified filtrate from cultures of *Vibrio cholerae*. It was supplied in an acetate buffer at pH 5·5 containing calcium ions and had a quoted strength of 500 units/ml. (Behringwerke).

### *Treatment of tumour cells*

Ascites fluid was withdrawn from a mouse 7 days after inoculation with tumour, spun down gently and washed 5 times in Hanks Balanced Salt Solution (HBSS). Tumour cells were incubated in either acetate buffer, in 500 units/ml. neuraminidase, or in neuraminidase previously heated to 60° C. for 30 minutes. The cells were incubated at 37° C. for 30 minutes and then washed again 5 times in HBSS. Tumour cell viability was assessed by lissamine green exclusion before and after incubation. The cells were then administered to 3 groups of mice. Each mouse received $4 \times 10^6$ tumour cells intraperitoneally and was observed for 60 days or until death occurred.

### *Hydrocortisone treated mice*

Two groups of mice were given daily subcutaneous injections of hydrocortisone sodium succinate as described by Wheatley and Easty (1964). For the first 3

days they were given 3 mg. each and 1 mg. per day thereafter. On the third day 1 group received intraperitoneal neuraminidase-treated tumour cells and the remaining group were given untreated cells which had been incubated in the acetate buffer.

*Attempted immunization of mice*

Spleen cells were obtained by filtering the chopped and minced spleens from 6 adult male $A_2G$ mice. These cells were washed in HBSS. Half of them were then incubated in 500 units/ml. neuraminidase for 30 minutes at 37° C. The supernatant was retained and the cells washed 5 times in HBSS. Landschütz ascites tumour cells were similarly treated and the supernatant retained. The recipient mice were divided into 7 groups and received one of the following intraperitoneal injections: $4 \times 10^6$ neuraminidase-treated tumour cells, 0·2 ml. treated tumour cell supernatant, 0·2 ml. neuraminidase solution (500 units/ml.), 0·2 ml. HBSS, $4 \times 10^6$ neuraminidase-treated $A_2G$ spleen cells, 0·2 ml. treated spleen cell supernatant, $4 \times 10^6$ $A_2G$ spleen cells. All cell suspensions were administered in 0·2 ml. HBSS. These mice were left for 14 days and then challenged by the intraperitoneal administration of $5 \times 10^4$ Landschütz ascites tumour cells per mouse. They were examined daily for the development of tumour.

*Rechallenge*

All mice which survived the intraperitoneal administration of treated cells and were clinically tumour free at 30 days were rechallenged by the intraperitoneal injection of $5 \times 10^4$ tumour cells ($5000 \times LD_{50}$ for this tumour) and observed for tumour development.

*Mouse sera*

Mice which survived tumour inoculation and control mice which were succumbing to tumour were bled by cardiac puncture and their sera separated and stored at −20° C. These sera were assayed for anti-tumour agglutinins using the slide technique described by Lindenmann (1964).

*Adsorption of antiserum*

0·5 ml. Aliquots of a 1 : 5 dilution high agglutinin antiserum were adsorbed by incubation for 1 hr with $20 \times 10^6$ tumour cells either neuraminidase-treated or untreated. After incubation the cells were separated by centrifugation and the supernatant antiserum re-assayed for agglutinins. Unadsorbed antiserum was assayed at the same time.

RESULTS

Viability of cells as judged by dye exclusion was approximately 85 per cent and was unaltered by incubation in neuraminidase or any of the control solutions.

The results are summarized in Tables I and II. Untreated tumour cells caused massive ascites in all injected control animals. Death occurred approximately 16 days after inoculation and was associated with respiratory distress and hypothermia. Haemoperitoneum was unusual in $A_2G$ mice but had been common in previous experiments using adult male CBA mice.

TABLE I.—*The Effect of Incubation of Landschütz Ascites Tumour Cells in Neuraminidase and Heat-Inactivated Neuraminidase on the Subsequent Development of Tumour in Normal and Hydrocortisone Treated Mice. Results are Expressed as Number of Mice Surviving After* 30 *days/number of Mice Injected.*

| | Treatment of recipient mice | |
|---|---|---|
| Treatment of Tumour Cells | Nil | Hydrocortisone |
| Nil | 0/6 | 0/6 |
| 500 units/ml. Neuraminidase | 6/6 Rechallenged* 6/6 | 0/6 |
| Heated neuraminidase 500 units/ml. | 0/6 | — |

* Animals rechallenged after 30 days with $5 \times 10^4$ tumour cells. Result expressed as survival after another 30 days.

TABLE II.—*The Effects of Pre-Exposure of Mice to Neuraminidase, Neuraminidase-Treated Tumour Cells, Treated Spleen Cells, Untreated Spleen Cells and their Supernatant Fluids on a Subsequent Challenge* (14 *days later*) *with* $5 \times 10^4$ *Landschütz Ascites Tumour Cells. Results are Expressed as Number of Mice Surviving After* 30 *days/number of Mice Injected.*

| Intraperitoneal injection | Result of challenge with $5 \times 10^4$ tumour cells |
|---|---|
| Neuraminidase treated tumour cells | 6/6 Rechallenged 6/6* |
| Treated tumour cell supernatant | 0/6 |
| Neuraminidase only | 0/6 |
| Neuraminidase treated $A_2G$ spleen cells | 0/6 |
| Treated spleen cell supernatant | 0/6 |
| Untreated $A_2G$ spleen cells | 0/6 |
| HBSS | 0/6 |

* Animals rechallenged after 30 days with $5 \times 10^4$ tumour cells. Result expressed as survival after another 30 days.

*Effect of neuraminidase on tumour development*

Mice receiving cells treated in acetate buffer only, all developed tumour and died at about Day 16. All animals which received neuraminidase-treated tumour cells remained tumour free at 60 days. Rechallenge of animals with 5000 $LD_{50}$ untreated tumour cells resulted in no tumour growth. Sacrifice and subsequent dissection of these animals revealed no evidence of tumour, either solid or ascitic.

Tumour cells incubated in previously heated neuraminidase produced tumour in all inoculated animals. Thus the effect of neuraminidase on the tumour cells is abolished by heating to 60° C. for 30 minutes.

*Hydrocortisone-treated mice*

When neuraminidase-treated tumour cells were injected into hydrocortisone-treated mice tumour developed in all recipients (Table I). The mean survival of these animals after inoculation was approximately 23 days as was the survival of hydrocortisone-treated mice given untreated tumour cells. Thus hydrocortisone treatment of the host mice abolished the effect of neuraminidase on the tumour.

*Anti-tumour agglutinins*

Using the slide agglutination technique powerful agglutinating activity was detected in the sera of all surviving mice. Titres as high as 1 : 5,120 were detected. In control animals no agglutinins were detected above 1 : 8.

*Adsorption of antisera*

The unadsorbed antiserum gave an agglutination titre of 1 : 1600. After adsorption for 1 hour with untreated tumour cells this titre fell to 1 : 200. Adsorption of the antiserum with an identical number of neuraminidase-treated tumour cells produced an identical fall in titre to 1 : 200. This indicates that neuraminidase treatment of the tumour cells did not modify their ability to adsorb agglutinins.

*" Immunization " experiments*

The results from this group of experiments are shown in Table II. A protective effect against subsequent challenge with untreated tumour cells could only be produced by inoculation of neuraminidase-treated washed tumour cells. Treated tumour cell supernatant, neuraminidase alone, HBSS, treated spleen cells and the supernatant from these cells produced no effect, indicating that neuraminidase induces tumour immunity by its effect on intact tumour cells.

### DISCUSSION

The effect of neuraminidase on the immunogenicity of tumour cells is poorly understood. There are many possible explanations of this phenomenon. The results of the present study, however, indicate that most of these are untenable.

*Release of immunogenic molecules*

Lindenmann and Klein (1967) have suggested that neuraminidase acts by releasing immunogenic molecules from the cell allowing them to be detected by the host's immune mechanisms. The supernatant from neuraminidase-treated tumour cells, however, does not immunize mice against the tumour under conditions where washed treated tumour cells induce a powerful immunity. The enzyme does not therefore allow the escape of antigens during the period of incubation by increasing the " leakiness " of the cell wall.

*Cross reacting antigens*

Neuraminidase is derived from bacterial cultures. It is not inconceivable that contaminating bacterial antigens might adsorb to the tumour cell surface during incubation and cross react with tumour antigens. Pre-exposure of the host mice to neuraminidase provides no protection against tumour growth. This indicates that the neuraminidase preparation used did not contain antigens which directly cross reacted with the tumour. Heat lability studies show that the neuraminidase preparation was inactivated at 60° C. in the presence of calcium ions. Similar studies of receptor destroying enzyme (RDE) reveal that the active principle has similar heat lability characteristics (Burnet and Stone, 1947). Further work is in progress to characterize the active component of the enzyme preparation used in the above experiments.

*Modification of Host isoantigens*

It could be argued that the enzyme acts on $A_2G$ strain isoantigens, modifying them so that they cross react with the tumour. However, pre-exposure to neuraminidase-treated $A_2G$ spleen cells affords no protection against tumour. It would also have to be postulated that neuraminidase is carried across to the host animal with the tumour cells. This would seem unlikely in view of the extensive washing of treated cells employed in this study.

*Non-specific stimulation of the immune response*

Sanford (1967) has shown that this explanation is unlikely by demonstrating that the addition of Freund's adjuvant to injected tumour cells induced no evidence of immunity to tumour growth. She also revealed that treatment of mice with neuraminidase did not affect first set skin graft rejection.

*Modification of tumour cell periphery*

In the present study neuraminidase-treated tumour cells grew in hydrocortisone-treated mice, demonstrating that the inoculated tumour cells were viable and capable of growing in the absence of an effective immune response. This study incidentally supports Wheatley and Easty's observation (1964) that tumour development is slowed in hydrocortisone-treated animals. Dye exclusion studies have also suggested that tumour cell viability is not affected by neuraminidase. Studies of the adsorption of agglutinating antisera indicate that neuraminidase treatment does not affect antigen-antibody interactions in an agglutination system. This would also indicate that neuraminidase does not affect the antigens *per se*, either quantitatively or qualitatively. In other words, it probably operates at the afferent end of the immunological arc. Its effect must therefore be directed to the periphery of the intact living tumour cell. Exposure to neuraminidase seems to allow previously undetected cell wall antigens to be detected by the host's immune mechanisms.

Neuraminidase acts on sialomucins by hydrolytic cleavage of O-glycoside bonds between the keto group of sialic (*N*-acetyl neuraminic) acid and its underlying amino-sugar (Gottschalk, 1957). Thus the unmasking of tumour antigens is associated with removal of the terminal sialic acid moiety from cell wall sialomucins. The free carboxyl groups of the sialic acid moiety are electronegative and make a substantial contribution to the negative charge at the periphery of many mammalian cell types. Thus the unmasking of tumour antigens by neuraminidase isa ssociated with a fall in electronegative charge at the cell periphery (Currie, 1967). This phenomenon could provide two possible explanations of the masking of antigens by cell wall sialomucins and for the effect of neuraminidase.

(a) As previously postulated (Currie, 1967) the high net negative charge at the cell periphery could directly inhibit the approach of negatively charged lymphoid cells by Coulomb forces. By reducing this charge neuraminidase would allow more stable intercellular contacts to occur and thus initiate the cellular component of the immune response.

(b) The sialomucins could mask antigens from detection by steric hindrance alone. Treatment with neuraminidase would reduce the negative charges at the free ends of the mucoprotein molecules and thus, as suggested by Gottschalk (1960), allow a rearrangement of the tertiary structure of the polypeptide component of these molecules. This rearrangement might abolish any masking due to steric hindrance.

Studies of the effects of neuraminidase on the surface charge of tumour cells and spleen cells have so far lent support to the former hypothesis (Currie, 1967).

The effect of neuraminidase on the immunogenicity of tumours has so far only been described in the Ehrlich, the Landschütz and the $TA_3$ ascites tumours. These are non-specific transplantable tumours whose antigenicity is probably derived from histocompatibility antigens of the mouse of origin of each tumour. Further studies of autochthonous tumours are in progress to determine whether tumour-specific antigens are masked by cell wall sialomucins.

Gasic and Beydak (1961) have demonstrated that many tumour cells possess an acidic mucoprotein coat and Gasic and Gasic (1962) have disrupted this layer with neuraminidase, demonstrating the presence of sialic acid. Studies of cell surface charge have also revealed that many animal tumours possess a high net negative charge (Ruhenstroth-Bauer *et al.*, 1962) which can be attributed wholly or in part to the presence of sialic acid in the cell periphery. Sialic acid containing mucoproteins are also present in the peritrophoblastic " fibrinoid " material. A recent study of the effects of neuraminidase on the immunogenicity of mouse trophoblast (Currie, van Doorninck and Bagshawe, 1968, unpublished data) has suggested that the histocompatibility antigens on mouse trophoblast are masked by pericellular sialomucins. Disruption of pericellular sialomucins around trophoblast and some experimental tumours is thus associated with the " unmasking " of previously hidden antigenicity and suggests that this form of specific afferent inhibition of the immune response (Billingham, Brent and Medawar, 1956) may play an important role in both foeto-maternal and tumour-host interactions.

Neuraminidase appears to be a valuable tool for the exploration of the role of the cell periphery in the immunology of such interactions.

### SUMMARY

The mode of action of neuraminidase on the immunogenicity of the Landschütz ascites tumour has been investigated. When neuraminidase-treated Landschütz ascites tumour cells were injected into $A_2G$ male mice no tumour growth occurred and a powerful tumour immunity ensued. Circulating anti-tumour agglutinins appeared in the peripheral blood and when rechallenged with untreated tumour, the mice remained tumour free. Treament of the recipient mice with hydrocortisone abolished this effect. It was also abolished by heating the neuraminidase preparation to 60° C. for 30 minutes in the presence of calcium ions. The neuraminidase preparation used did not contain cross-reacting antigens and it did not induce cross reaction of host cell isoantigens with the tumour. The supernatant from treated tumour cells was not immunogenic indicating that antigenic molecules were not released from the tumour cell surface by treatment with this enzyme. Cell viability was not affected by neuraminidase. The weight of evidence implies that this enzyme works by a direct effect on the cell periphery.

The role of cell wall sialomucins in the masking of antigens and the action of neuraminidase on this mechanism are discussed.

G. A. Currie gratefully acknowledges a Saltwell Scholarship from the Royal College of Physicians, London.

Studies in these Laboratories are supported by the British Empire Cancer Campaign for Research and the Charing Cross Hospital Research Sub-Committee.

### REFERENCES

Billingham, R. E., Brent, L. and Medawar, P. B.—(1956) *Transplantn Bull.*, **3**, 84.
Burnet, F. M. and Stone, J. D.—(1947) *Aust. J. exp. Biol. med. Sci.*, **25**, 227.
Currie, G. A.—(1967) *Lancet*, ii, 1336.
Currie, G. A. and Bagshawe, K. D.—(1967) *Lancet*, i, 708.
Gasic, G. and Beydak, T.—(1961) ' Biological Interactions in Normal and Neoplastic Growth ', edited by Brennan, M. J. and Simpson, W. L. London (Churchill).
Gasic, G. and Gasic, T.—(1962) *Proc. natn. Acad. Sci. U.S.A.*, **46**, 1172.
Gottschalk, A.—(1960) *Nature, Lond.*, **186**, 949.—(1957) *Biochim. biophys. Acta.*, **23**, 645.
Lindenmann, J.—(1964) *J. Immun.*, **92**, 912.
Lindenmann, J. and Klein, P. A.—(1967) ' Immunological Aspects of Viral Oncolysis '. Berlin (Springer-Verlag).
Ruhenstroth-Bauer, G., Fuhrmann, G. F., Kubler, W., Rueff, F. and Monk, K.—(1962) *Z. Krebsforsch.*, **65**, 37.
Sanford, B. H.—(1967) *Transplantation*, **5**, 1273.
Wheatley, D. N. and Easty, G. C.—(1964) *Br. J. Cancer*, **18**, 743.